# Sleep and Serotonin Modulate Paracapsular Nitric Oxide Synthase Expressing Neurons of the Amygdala

**DOI:** 10.1523/ENEURO.0177-16.2016

**Published:** 2016-10-05

**Authors:** Marco Bocchio, Simon P. Fisher, Gunes Unal, Tommas J. Ellender, Vladyslav V. Vyazovskiy, Marco Capogna

**Affiliations:** 1 MRC Brain Network Dynamics Unit, Department of Pharmacology, University of Oxford, Oxford OX1 3TH, UK; 2 Department of Physiology, Anatomy and Genetics, University of Oxford, Oxford OX1 3PT, UK; 3 Department of Pharmacology, University of Oxford, Oxford OX1 3QT, UK; 4 Department of Biomedicine, Aarhus University, 8000 Aarhus C, Denmark; 5 The Danish Research Institute of Translational Neuroscience (DANDRITE), Nordic EMBL Partnership for Molecular Medicine, Aarhus University, 8000 Aarhus C, Denmark

**Keywords:** amygdala, electrophysiology, interneuron, nitric oxide, serotonin, sleep

## Abstract

Unraveling the roles of distinct neuron types is a fundamental challenge to understanding brain function in health and disease. In the amygdala, a brain structure regulating emotional behavior, the diversity of GABAergic neurons has been only partially explored. We report a novel population of GABAergic amygdala neurons expressing high levels of neuronal nitric oxide synthase (nNOS). These cells are predominantly localized along basolateral amygdala (BLA) boundaries. Performing *ex vivo* patch-clamp recordings from nNOS^+^ neurons in Nos1-Cre^ER^;Ai9 mice, we observed that nNOS^+^ neurons located along the external capsule display distinctive electrophysiological properties, axonal and dendritic arborization, and connectivity. Examining their c-Fos expression, we found that paracapsular nNOS^+^ neurons are activated during a period of undisturbed sleep following sleep deprivation, but not during sleep deprivation. Consistently, we found that dorsal raphe serotonin [5-hydroxytryptamine (5-HT)] neurons, which are involved in sleep–wake regulation, innervate nNOS^+^ neurons. Bath application of 5-HT hyperpolarizes nNOS^+^ neurons via 5-HT1A receptors. This hyperpolarization produces a reduction in firing rate and, occasionally, a switch from tonic to burst firing mode, thereby contrasting with the classic depolarizing effect of 5-HT on BLA GABAergic cells reported so far. Thus, nNOS^+^ cells are a distinct cell type of the amygdala that controls the activity of downstream neurons in both amygdaloid and extra-amygdaloid regions in a vigilance state-dependent fashion. Given the strong links among mood, sleep deprivation, and 5-HT, the recruitment of paracapsular nNOS^+^ neurons following high sleep pressure may represent an important mechanism in emotional regulation.

## Significance Statement

Understanding the function of GABAergic neurons of the amygdala can greatly improve our knowledge of the cellular underpinnings of emotional behavior and improve therapies for psychiatric disorders. Here we report a novel GABAergic neuron type of the BLA that displays high levels of neuronal nitric oxide synthase. This neuron type shows high or low early gene expression during sleep or wakefulness, respectively. Our data suggest that reduced recruitment of these cells during sleep deprivation could originate, at least in part, from their inhibition by 5-HT, which is preferentially released during wakefulness but not during sleep. This work provides an important link between a specific GABAergic cell type of the amygdala, a wake-promoting neuromodulator, and the sleep–wake cycle.

## Introduction

The presence of functionally heterogeneous GABAergic neurons equips the brain with unparalleled computational power (Klausberger and Somogyi, 2008; Hangya et al., 2014). Deciphering the operations carried out by distinct classes of inhibitory cells is considered one of the major neurobiological challenges (Lovett-Barron and Losonczy, 2014).

The basolateral amygdala (BLA) is a cortical-like brain region controlling emotional behavior (Duvarci and Pare, 2014; Janak and Tye, 2015). Compared to hippocampus or neocortex, our knowledge of anatomy, physiology and role in behavior of specific GABAergic populations in the rodent BLA is limited (Capogna, 2014). From a functional perspective, two inhibitory neuron classes have received particular attention so far: the parvalbumin (PV)-expressing and the somatostatin (SOM)-expressing interneurons (Rainnie et al., 2006; Woodruff and Sah, 2007b; Wolff et al., 2014).

Many other GABAergic neuron types have been detected in the BLA of rodents (Spampanato et al., 2011), but our understanding of their functional roles is scant. In addition to the expression of PV and SOM, BLA interneurons express a variety of neurochemical markers, such as calbindin (McDonald and Mascagni, 2001; Bienvenu et al., 2012), calretinin (McDonald and Mascagni, 2001), vasoactive intestinal peptide (Mascagni and McDonald, 2003), cholecystokinin (Jasnow et al., 2009; Vogel et al., 2016), and neuronal nitric oxide synthase (nNOS; McDonald et al., 1993; Usunoff et al., 2006). Neurons of the BLA expressing nNOS represent a particularly intriguing cell populations for several reasons. First, in areas with interneuron diversity similar to the BLA, such as hippocampus or neocortex, nNOS^+^ neurons are as abundant, or even denser, than PV^+^ and SOM^+^ cells (Fuentealba et al., 2008; Tricoire and Vitalis, 2012), suggesting a prominent impact on both hippocampal and cortical circuits. Second, they are able to modulate neurons through a variety of mechanisms, including slow inhibition (Capogna and Pearce, 2011), retrograde release of nitric oxide (Li et al., 2014), and potentially via other neuropeptides (Fuentealba et al., 2008; Tricoire and Vitalis, 2012), suggesting they might fulfill a function that is different from those of other classic interneuron types. Third, neocortical nNOS^+^ neurons coexpressing SOM and NPY are thought to be atypical long-range GABAergic projection neurons (Tomioka et al., 2005; Tamamaki and Tomioka, 2010), and this might also apply to the BLA (McDonald et al., 2012; McDonald and Zaric, 2015). Despite their prominence, the physiological and behavioral roles of GABAergic nNOS^+^ neurons of the BLA remain elusive.

The activity of BLA GABAergic neurons is eminently controlled by subcortical neuromodulators released during arousal, such as serotonin [5-hydroxytryptamine (5-HT)], acetylcholine, and noradrenaline (Tully et al., 2007; Bocchio et al., 2015; Unal et al., 2015a). Among those, 5-HT neurotransmission is compelling because it modulates emotional learning in the BLA (Bocchio et al., 2016), but it is also involved in the sleep–wake cycle (Portas et al., 2000; Gao et al., 2002). Specifically, extracellular forebrain 5-HT levels are low during non-rapid eye movement (NREM) and rapid eye movement (REM) sleep, and high during wakefulness (Portas et al., 1998; Bjorvatn et al., 2002). Consistently, electrophysiological experiments have shown that dorsal raphe nuclei (DRN) 5-HT neurons fire at higher rates during wakefulness and at lower rates during NREM sleep, and are virtually silent during REM sleep (Sakai, 2011). Since the activity of BLA neurons also follows the sleep–wake cycle (Paré and Gaudreau, 1996), 5-HT could play a crucial role in the vigilance state-dependent activity of BLA cells.

At a cellular level, the most commonly established effect of 5-HT in the BLA is the depolarization of GABAergic interneurons (Rainnie, 1999), and among those of PV^+^ interneurons via 5-HT2A receptors (Bocchio et al., 2015). However, recent *in situ* hybridization data have shown that BLA NPY^+^ cells, some of which are thought to be nNOS^+^ (McDonald et al., 1993), can also express inhibitory 5-HT1A receptors (Bonn et al., 2013), suggesting that 5-HT could also hyperpolarize some GABAergic cells. Defining the diversity of 5-HT actions on BLA neuron types is crucial if we are to understand the cellular dynamics occurring in the BLA across different brain states.

In this study, we aimed to functionally characterize nNOS^+^ neurons of the mouse BLA and to shed light on their behavioral role. Additionally, we wished to probe whether the 5-HT modulation of nNOS^+^ neurons is in line with the action of 5-HT on previously characterized GABAergic neurons, and whether this modulation is consistent with the behavioral recruitment of nNOS^+^ neurons.

## Materials and Methods

### Animals

Since nNOS is broadly expressed during development (Bredt and Snyder, 1994), but its expression is more restricted to particular cells following postnatal day 15 (P15; Kubota et al., 2011; Taniguchi et al., 2011), an inducible Cre driver line (Nos1-Cre^ER^; B6;129S-*Nos1^tm1.1(cre/ERT2)Zjh^*/J; stock #014541, The Jackson Laboratory) was used to elicit Cre recombination postnatally. Nos1-Cre^ER+/−^ mice were crossed with Ai9^+/+^ reporter mice (B6.Cg-*Gt(ROSA)26Sor^tm9(CAG-tdTomato)Hze^/J*; stock #007909, The Jackson Laboratory) to generate Nos1-Cre;Ai9 offspring. To quantify the overlap of neurochemical markers, WT C57BL/6J mice (Charles River Laboratories) were used. For anterograde tracing experiments, SERT-Cre^+/−^ mice (MMRRC, B6.Cg-Tg(Slc6a4-cre)Et33Gsat; stock #031028-113, UC Davis) were used. Mice were housed with their littermates with *ad libitum* access to food and water in a dedicated housing room with a 12 h light/dark cycle. To induce Cre recombinase and label nNOS^+^ neurons with tdTomato, Nos1-Cre^ER^;Ai9 mice (age range, postnatal day 20–45) received one to three intraperitoneal injections of tamoxifen (10 mg/ml in corn oil, 10 μl/g body weight/d). For patch-clamp and anatomical experiments, mice (age range, postnatal day 27–60) were used at least 1 week after the first tamoxifen injection.

For sleep experiments, adult male C57BL/6J mice (15 weeks of age) were individually housed in custom-made clear Plexiglas cages (20.3 × 32 × 35 cm) with free access to a running wheel and *ad libitum* food and water. Cages were housed in ventilated, sound-attenuated Faraday chambers (two cages per chamber; Campden Instruments) under a standard 12 h light/dark cycle [lights on 8:00 A.M., zeitgeber time 0 (ZT0); light levels, ∼120–180 lux]. Room temperature (RT) and relative humidity were maintained at 22 ± 1°C and 50 ± 20%, respectively. Mice were habituated to both the cage and recording cables for a minimum of 16 d prior to recording.

All procedures involving experimental animals were performed in compliance with the Animals (Scientific Procedures) Act, 1986 (UK) and associated regulations, under approved project licenses by Home Office UK (30/3061 and 70/7483) and with Society for Neuroscience Policies on the Use of Animals in Neuroscience Research.

### *Ex vivo* recordings

Nos1-Cre^ER^;Ai9 mice (age range, postnatal day 27–60) were decapitated under deep isoflurane anesthesia (4% in O2), and their brains were rapidly removed and placed in ice-cold sucrose-containing artificial CSF (ACSF) cutting solution containing the following (in mm): 75 sucrose, 87 NaCl, 25 NaHCO3, 2.5 KCl, 1.25 NaH2PO4, 0.5 CaCl2, 7 MgCl2, and 25 glucose, saturated with 95% O2, 5% CO2, at pH 7.3–7.4. Slices (325 μm thickness), including the amygdala were cut (Microm HM 650 V, Thermo Fisher Scientific) and transferred onto a nylon mesh where they were maintained in a chamber initially containing sucrose ACSF cutting solution at 37°C for 30 min. During this period, the cutting solution was gradually substituted (5 ml/min) with normal ACSF consisting of the following (in mm): 130 NaCl, 24 NaHCO3, 3.5 KCl, 1.25 NaH2PO4, 2.5 CaCl2, 1.5 MgSO4, and 10 glucose, saturated with 95% O2, 5% CO2, at pH 7.3.

Slices were transferred to a submerged recording chamber and continuously perfused with oxygenated ACSF at a rate of ∼5 ml/min and at a mean temperature of 34 ± 1°C. Neurons were visualized with an upright Axioskop microscope (Zeiss) using phase-contrast microscopy under a LUMPlanFI 60× immersion objective (Olympus). A mercury vapor short-arc lamp (100 W; N HBC 103, Zeiss) was connected to the epifluorescence system to visualize the tdTomato^+^ neurons. Micropipettes (5–6 MΩ) were pulled from borosilicate glass capillaries (1.2 mm; GC120F, Harvard Apparatus) with a DMZ puller (Zeitz-Instrumente). Somatic whole-cell patch-clamp recordings were performed from visually identified tdTomato^+^ neurons. Electrodes were filled with an intracellular solution composed of the following (in mm): 126 K-gluconate, 4 KCl, 4 ATP-Mg, 0.3 GTP-Na2, 10 Na2-phosphocreatine, 10 HEPES, and 0.2-0.4% biocytin, with osmolarity of 270–280 mOsmol/L without biocytin, at pH 7.3 adjusted with KOH.

For paired recordings, the presence of a connection was tested by evoking an action current (3-ms-long voltage step from −60 to 0 mV) in paracapsular nNOS (pc-nNOS) cells. In some cases (*n* = 3), nearby cells were loaded with the same intracellular solution mentioned above (Cl- reversal potential, E_Cl_, −91 mV) and held in voltage-clamp mode at −40 mV. The remaining cells (*n* = 16) were loaded with an intracellular solution with higher Cl^−^ to increase the driving force of IPSCs (E_Cl_, −12 mV). This solution consisted of the following (in mm): 42 K-gluconate, 84 KCl, 4 ATP-Mg, 0.3 GTP-Na2, 10 Na2-phosphocreatine, 10 HEPES, and 0.2–0.4% biocytin, with osmolarity of 270–280 mOsmol/L without biocytin, pH 7.3 adjusted with KOH. In these cases, nearby cells were held at −65 mV. Since this resulted in inward polarity of Cl^−^ currents, glutamatergic transmission was blocked with either 3 mm kynurenic acid (or 10 μm NBQX and 50 μm D-APV) to isolate GABAergic IPSCs. Action currents were evoked at least 10 times, with 20 s interval between sweeps. Principal neurons were distinguished from interneurons according to the following parameters: (1) smaller fast afterhyperpolarization (fAHP) amplitude and prominent medium AHP in an instantaneous firing rate protocol; (2) adapting, <20 Hz maximum firing rates; (3) lower input resistance (R_in_; <150 MΩ); and (4) longer spike half-width (∼1 ms). Electrophysiological signals were amplified using an EPC9/2 amplifier (HEKA Electronik) and acquired using Patchmaster software (HEKA Electronik). Recordings were accepted only when the initial seal resistance was >2 GΩ, the holding current necessary to clamp the cell at −60 mV was smaller than −50 pA, and the series resistance did not change by >20% throughout the experiment. No correction was made for the liquid junction potential (16 mV) between the pipette and the ACSF.

Membrane potential (*V*_m_) during 5-HT application was monitored while holding neurons in current clamp at −60 ± 2 mV. Hyperpolarizing and depolarizing current steps were injected every 10 s to monitor R_in_ and firing, respectively. At the end of the recording, some slices containing biocytin-filled cells were fixed overnight at 4°C in 4% paraformaldehyde (PFA) and 15% saturated picric acid in 0.1 m PB. After 24 h, slices were embedded in gelatin and re-sectioned into 60- to 80-μm-thick sections with a VT-1000 vibrating microtome (Leica).

### Analysis of *ex vivo* recordings

Analysis of synaptic currents and intrinsic membrane properties was performed using IGOR Pro (WaveMetrics) and MATLAB (MathWorks). The R_in_ was calculated from the slope of steady-state voltage responses to a series of 8–10 subthreshold current injections (from −30 to +60 pA) lasting 400 ms. The AHP (in mV) was determined from the first spike in response to a juxtathreshold positive current injection. The spike duration of the action potential was measured as the width at half-amplitude between the threshold potential and the peak of the action potential, which was evoked by a strong (800–1000 pA) and short (2–5 ms) depolarizing current pulse. The membrane time constant τ was estimated from the monoexponential curve fitting of voltage responses to a −30 pA hyperpolarizing pulse. The rheobase (in pA) was determined as a 50 ms current injection, able to generate a spike in 50% of the cases in 10 trials. The instantaneous firing rate (in Hz) was defined as the number of action potentials evoked during a 1 s depolarizing current pulse of twice the amplitude of the rheobase current. The membrane capacitance was calculated as the ratio between the time constant and the R_in_. The adaptation index (range, 0–1) was defined as the ratio between the first and last interspike intervals (ISIs; in ms) elicited by the same pulse used to measure the instantaneous firing rate. The resting *V*_m_ was estimated by averaging a 20 s current-clamp trace recorded at a 0 pA holding current. Although many nNOS^+^ neurons were spontaneously active, spikes did not contaminate this estimate because firing rates were <5 Hz and the average of the 20 s trace matched the *V*_m_ sampled during ISIs.

To minimize artificial changes in firing rate due to recording conditions, cell-attached recordings were performed in loose-patch configuration (<50 MΩ seal). Spikes were acquired in voltage clamp by setting the pipette potential to obtain 0 pA of membrane current (Alcami et al., 2012). Neurons were defined as “bursting” during 5-HT application if the peak of the ISI histogram (in Log scale) was <100 ms.

### Electroencephalogram recordings and sleep deprivation

#### Surgical procedures and electrode implantation

Surgical procedures were performed using aseptic techniques under isoflurane anesthesia (3–5% induction, 1–2% maintenance) and Metacam (1–2 mg/kg, s.c.; Boehringer Ingelheim) was administered preoperatively. During surgery, animals were head fixed using a stereotaxic frame (David Kopf Instruments) and liquid gel (Viscotears, Alcon Laboratories) was applied to protect the eyes. In all animals, electroencephalogram (EEG) screws were placed in the frontal [motor area: anteroposterior (AP), +2 mm; mediolateral (ML), +2 mm] and occipital (visual area, V1: AP, −3.5/−4 mm; ML, +2.5 mm) cortical regions using procedures previously described (Cui et al., 2014). A reference screw electrode was placed above the cerebellum, and an additional anchor screw was placed in the left parietal hemisphere to ensure implant stability. EEG screws were soldered (prior to implantation) to custom-made headmounts (Pinnacle Technology), and all screws and wires were secured to the skull using dental acrylic. Two single-stranded, stainless steel wires were inserted on either side of the nuchal muscle to record electromyography (EMG). Saline (0.1 ml/20 g body weight, s.c.) was administered postoperatively and animals were provided thermal support throughout and following surgery. Metacam (1–2 mg/kg) was orally administered for at least 3 d after surgery. A minimum 2 week recovery period was permitted prior to cabling the animals.

#### Experimental design

On the experimental day, following a stable 24 h baseline recording, mice were divided into the following two groups: sleep deprivation (SD, *n* = 4); and SD plus recovery sleep (RS; *n* = 4). RS was defined as the sleep opportunity occurring immediately following SD, and was limited to 1.5–2 h (Morairty et al., 2013). In both groups, SD was performed in the home cage of the animal for a continuous 4 h period starting at light onset. During this time, animals were spontaneously awake, and their behavior as well as their EEG/EMG recordings were under constant visual observation. Sleep was prevented by regularly providing the animals with novel objects, an effective method that mimics natural conditions of wakefulness, is ethologically relevant, and does not appear to stress the animals (Palchykova et al., 2006; Vyazovskiy et al., 2007). All mice were well habituated to the experimenter and to the exposure to novel objects prior to the experiment. Novel objects included nesting and bedding material from other cages, wooden blocks, paper boxes, and tubes of different shape and color. SD was successful with 97.98 ± 2.51% of time spent awake during the 4 h procedure. After completion of the SD experiment, animals in the SD group were injected with an overdose of Euthatal (pentobarbitone sodium 200 mg/ml; 0.3 ml, i.p.), and upon the loss of a response to toe pinch were perfused transcardially with 30 ml of 0.9% PBS followed by 50 ml of 4% PFA in 0.1 m PB. All mice were perfused within ∼30 min after the end of 4 h of SD, and were kept awake continuously until the moment of injection with Euthatal. The mice in the SD+RS group were allowed to sleep undisturbed for a period of 1.5–2 h (an average of 1.81 ± 0.15 h spent in NREM and REM sleep) and then were perfused according to the same procedure. Special care was taken to ensure that the animals in the SD+RS group were not awake for longer than a few minutes prior to the injection of Euthanal. Brains were removed, postfixed in 4% PFA (in 0.1 m PB) overnight at 4°C, then thoroughly washed in PBS and left in 0.1 m PB plus 0.05% sodium azide until further processing for c-Fos/nNOS immunohistochemical analysis (see below). c-Fos protein is a marker of neuronal activation that is produced 30–60 min following stimulus/behavior onset (Sheng and Greenberg, 1990; Morgan and Curran, 1991). Several lines of evidence indicate that c-Fos levels can rapidly increase and decrease during both wake and sleep (Basheer et al., 1997; Cirelli and Tononi, 2000; Gerashchenko et al., 2008). These aspects render c-Fos staining a convenient approach to investigate effects of sleep and waking on neuronal activity (Cirelli and Tononi, 2000).

#### EEG recordings and power spectra analysis

Data acquisition was performed using the Multichannel Neurophysiology Recording System (TDT). Cortical EEG was recorded from frontal and occipital derivations. EEG/EMG data were filtered between 0.1 and 100 Hz, amplified (PZ5 NeuroDigitizer Preamplifier, TDT) and stored on a local computer at a sampling rate of 256.9 Hz. EEG/EMG data were resampled off-line at a sampling rate of 256 Hz. Signal conversion was performed using custom-written MATLAB scripts and was then transformed into European Data Format using open source Neurotraces software (www.neurotraces.com). For each recording, EEG power spectra were computed by a fast Fourier transform routine for 4 s epochs (using a Hanning window), with a 0.25 Hz resolution (SleepSign Kissei Comtec Co.). Vigilance states were scored off-line through manual visual inspection of consecutive 4 s epochs (SleepSign, Kissei Comtec Co.). Two EEG channels (frontal and occipital) and EMG were displayed simultaneously to aid vigilance state scoring. Vigilance states were classified as waking (low-voltage, high-frequency EEG with a high level of phasic EMG activity), NREM sleep (presence of slow waves, EEG signal of a high amplitude and low frequency), or REM sleep (low-voltage, high-frequency EEG with a low level of EMG activity). Great care was taken to eliminate epochs contaminated by eating, drinking, or gross movements resulting in artifacts in at least one of the two EEG derivations.

### Anterograde tracing

To selectively label dorsal raphe 5-HT axons, the viral vector AAV2-EF1a-DIO EYFP (UNC Vector Core, University of North Carolina, Chapel Hill, NC) was stereotaxically injected (1 μl at 100 nl/min) into the dorsal raphe nuclei [coordinates according to bregma and the brain surface (in mm): AP, −4.1; dorsoventral, −2.5, −2.2, −1.9] of SERT-Cre mice (age range, P30 to P75) anesthetized using 1–2% isoflurane in oxygen (2 L/min). On recovery from surgery, mice were administered buprenorphine 0.3 mg/kg, s.c., for postoperative analgesia. Three weeks were allowed for anterograde tracing before fixation by perfusion.

### Histological procedures

#### Immunohistochemistry

For quantification of the overlap between neurochemical markers, mice were transcardially perfused with saline followed by 4% PFA, 15% saturated picric acid in 0.1 m PB. Brains were sectioned using a vibratome (VT 1000 S, Leica) into 60-μm-thick slices. Sections were stored in 0.1 m PB containing 0.05% sodium azide until further usage. Resectioned slices (60–80 μm thickness) containing recorded and biocytin-filled neurons were incubated overnight at 4°C in 1:2000 Alexa Fluor 488-conjugated streptavidin (Invitrogen). Following blocking with 10% normal donkey serum for 1 h at RT, sections were incubated overnight at 4°C with the following primary antibodies: anti-5-HT raised in rabbit (1:2500; provided by H. Steinbusch, Maastricht University, Maastricht, The Netherlands); anti-c-Fos raised in rabbit (1:500; catalog #ab7963, Abcam); anti-GFP raised in chicken (1:1000; catalog #GFP-1020, Aves Labs); anti-nNOS raised in goat (1:500; catalog #ab1376, Abcam); anti-NK1 (substance P receptor) raised in rabbit (1:1000; AB5060, Chemicon); anti-somatostatin raised in rat (1:250; catalog #MAB354, Chemicon); and anti-vesicular GABA transporter (VGAT) raised in rabbit (1:500; provided by M. Watanabe, Frontier Institute Co. Ltd., Hokkaido, Japan; http://www.frontier-institute.com). Following 3× washes in PBS, immunoreactivity was revealed with Alexa Fluor 488- (1:500), DyLight Cy3- (1:500), or Alexa Fluor 647-conjugated (1:250) secondary antibodies (all raised in donkey; Jackson ImmunoResearch). For negative controls, the primary antibody was routinely omitted from the staining procedure with no positive fluorescence signal detected. In some cases, each secondary antibody was omitted in turn to confirm its specificity. Nissl staining was obtained via incubation in NeuroTrace 640/660 Deep-Red Fluorescent Nissl Stain (1:200; catalog #N-21483, Thermo Fisher).

All reagents were diluted in PBS containing 0.3% Triton X-100. Immunoreactivity was visualized using an epifluorescence microscope (AxioImager M2, Zeiss) or a laser-scanning confocal microscope (LSM 510, Zeiss). The boundaries between nuclei were determined with bright-field microscopy or Nissl staining.

#### Quantification of overlap between neurochemical markers

Sections containing the BLA of SD and SD+RS mice were imaged with the epifluorescence microscope mentioned above and StereoInvestigator software (MBF Bioscience). A region of interest delineating either the BLA or the external capsule next to the lateral amygdala (LA) was defined using a bright-field microscope under a 5× 0.16 numerical aperture (NA) objective lens. For quantification of neurochemical markers expressed by nNOS cells, stereological sampling was performed in both hemispheres from one of three sections in the range −0.8 to −2.2 mm from bregma. Series of tiled stacked images were acquired using a 40× 1.3 NA oil-immersion objective and 1 μm steps at a depth of 2–22 μm (“optical sections”) from the upper surface of each section. In order to minimize artifacts arising from surface irregularities, the first 2 μm from the upper surface were defined as the “guard zone” and were not scanned. Counting was performed off-line in StereoInvestigator. A neuron was counted only if its immunopositive nucleus came into focus in the optical section. Nuclei already in focus at the top optical section were not counted (West, 1999). For quantification of the overall percentage of c-Fos^+^ cells in the paracapsular area, stereological counting was performed by sampling three evenly spaced sections in the range −0.8 to −2.2 mm from bregma. To ensure the quantification of neuronal c-Fos, Nissl staining was used. Only nuclear c-Fos expression in Nissl-stained cells with a diameter of >10 μm was quantified. The experimenter was blind to behavioral testing conditions. Data were exported to Excel (Microsoft) and pooled for further analysis.

#### Neurolucida reconstruction

Two-dimensional drawings were performed for two biocytin-filled cells to reveal dendrites and axonal arborization present in the 325-μm-thick slice. The 60- to 80-μm-thick sections were processed with DAB using a previously published protocol (Unal et al., 2015b). Drawings were made using Neurolucida software (MBF Bioscience) under a light microscope (100× objective). Final drawings were corrected for tissue shrinkage caused by Triton X-100 processing. Dendritic and axonal lengths were calculated using the same software.

#### Statistical testing

Data are presented as the means ± SEM. Distributions were compared using Student’s *t* tests or one-way ANOVAs with Bonferroni *post hoc* correction. Statistical analysis was performed with GraphPad Prism (GraphPad Software) and SigmaPlot (Systat Software Inc.), where *p* < 0.05 was considered to be statistically significant.

#### Drugs

Serotonin hydrochloride, WAY 100635 maleate, NBQX, D-APV, and SR95531 were purchased from Tocris Bioscience. Kynurenic acid and tamoxifen were purchased from Sigma-Aldrich.

## Results

### Neurochemical profile of nNOS^+^ type I neurons of the BLA

**Figure 1.**
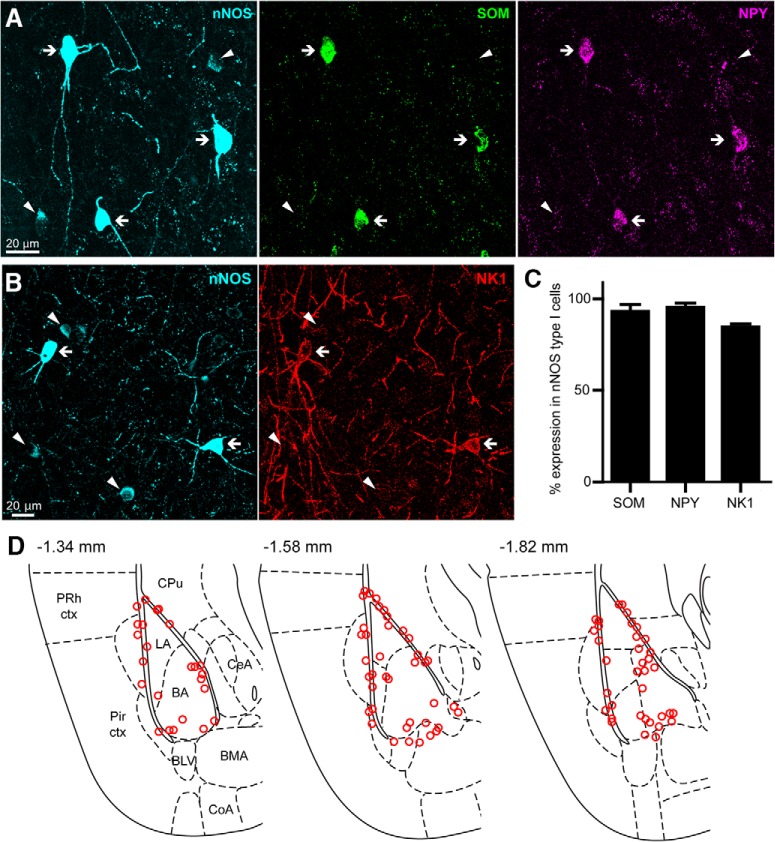
Neurochemical profile and localization of nNOS^+^ type I neurons of the BLA. ***A***, Confocal stack (*z*-stack, 27 μm) showing colocalization of SOM and NPY in BLA neurons with strong nNOS expression (nNOS^+^ type I, arrows) and no SOM and NPY immunoreactivity in neurons with weak nNOS expression (nNOS^+^ type II, arrowheads). ***B***, Confocal stack (*z*-stack, 13 μm) showing colocalization of NK1 in BLA nNOS^+^ type I neurons (arrows) and no NK1 immunoreactivity in nNOS^+^ type II neurons (arrowheads). ***C***, 93.2 ± 6.5% of nNOS^+^ type I neurons coexpressed SOM (*n* = 3 brains), 95.4 ± 5.4% coexpressed NPY (*n* = 6 brains), and 84.6 ± 3.1 coexpressed NK1 (*n* = 3 brains). ***D***, Three coronal sections illustrating the distribution of BLA nNOS^+^ type I neurons at different rostrocaudal positions. nNOS^+^ type I cells plotted at each level were mapped by collapsing three neighboring 60-μm-thick sections. Data are presented as the mean ± SEM. BLV, Basolateral ventral amygdala; BMA, basomedial amygdala; CeA, central amygdala; CoA, cortical amygdala; CPu, caudate–putamen; Pir ctx, piriform cortex; PRh, perirhinal cortex.

We aimed to uncover the anatomical and physiological features of GABAergic nNOS^+^ neurons of the BLA. First, we immunolabeled mouse coronal brain sections containing the amygdala for nNOS. We detected neurons with strong nNOS expression and others with light immunoreactivity (Fig. 1*A*), suggesting that nNOS^+^ neurons of the BLA can be classified according to the intensity of nNOS expression, as in the case of neocortex (Yan et al., 1996; Smiley et al., 2000; Lee and Jeon, 2005). Following previously used nomenclature (Yan et al., 1996; Perrenoud et al., 2012), we refer to neurons with strong nNOS expression as “type I” nNOS^+^ cells, and to neurons with weak nNOS expression as “type II” nNOS^+^ cells. Type I neurons displayed large ovoid somata and bitufted dendrites, whereas type II neurons had more heterogeneous soma size and dendritic emissions.

In cortical areas, type I nNOS^+^ cells often coexpress other SOM, neuropeptide Y (NPY), and neurokinin 1 (NK1) receptor. To investigate whether this coexpression pattern also applies to the BLA, we examined the proportion of type I and type II nNOS^+^ cells expressing these three markers. We found that 93 ± 4% of nNOS^+^ type I cells coexpressed SOM (*n* = 3 brains), 95 ± 2% coexpressed NPY (*n* = 6 brains), and 85 ± 2% coexpressed NK1 (*n* = 3 brains; Fig. 1*A–C*). In contrast, nNOS^+^ type II cells were mostly devoid of these neurochemical markers, with 2 ± 2% coexpressing SOM (*n* = 3 brains), 9 ± 3% coexpressing NPY (*n* = 6 brains), and none coexpressing NK1 (*n* = 3 brains; data not shown).

Thus, intense nNOS labeling identifies a neurochemically homogeneous population of BLA neurons. Since observations of neocortical nNOS^+^ neurons have demonstrated that type II cells are more heterogeneous and comprise several cell types (Tricoire and Vitalis, 2012), further investigations were focused on nNOS^+^ type I neurons. The latter cells were localized primarily along BLA borders, namely adjacent to the external capsule, intermediate capsule, and the border between basal (BA) and basomedial nuclei (Fig. 1*D*), which is in line with previous reports of nNOS^+^ “border cells” (McDonald et al., 1993; Usunoff et al., 2006). Importantly, type I neurons represented the great majority of cells expressing SOM and NPY (98.4 ± 1.6%, *n* = 2 brains).

### Intrinsic electrophysiological properties of pc-nNOS neurons

To study the physiology of nNOS type I neurons of the BLA, we crossed an inducible Cre driver mouse line (Nos1-Cre^ER^; Taniguchi et al., 2011) with an Ai9 reporter line. This enabled specific expression of tdTomato in nNOS^+^ neurons (97.9 ± 0.7% nNOS/tdTomato overlap; *n* = 3 brains), because neurons that express nNOS only transiently during development were not labeled with tdTomato. To selectively target nNOS^+^ type I neurons, we prepared acute coronal brain slices and performed whole-cell patch-clamp recordings from tdTomato^+^ cells located along the external capsule that separates the LA from the endopiriform claustrum/piriform cortex (in the following, called pc-nNOS^+^ neurons). In this region, type I cells constitute 80 ± 3% (*n* = 3 brains) of nNOS^+^ neurons and are easily distinguishable from type II cells due to their significantly larger ovoid somata (area, 128 ± 5 vs 88 ± 2 μm^2^; *p* < 0.0001; *n* = 25 cells from six brains).

**Figure 2.**
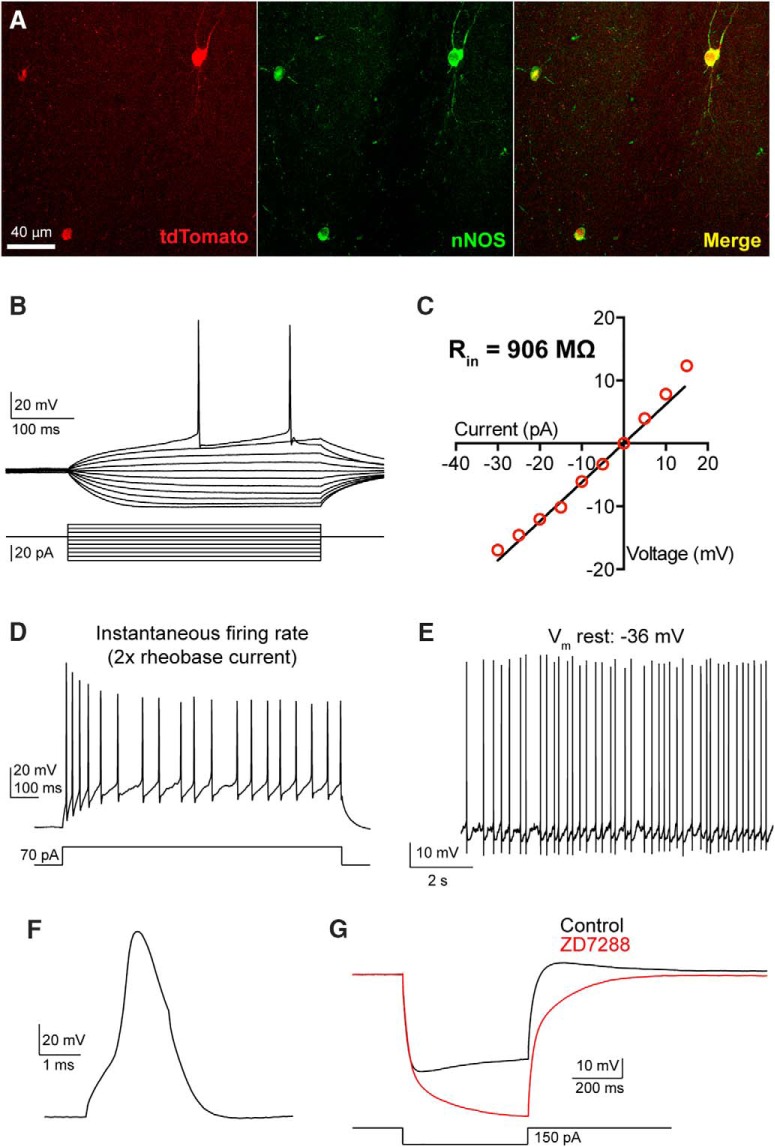
Electrophysiological properties of pc-nNOS neurons. ***A***, Colocalization of tdTomato and nNOS in BLA neurons from a Nos1-Cre^ER^;Ai9 mouse. ***B***, ***C***, Voltage responses to hyperpolarizing–depolarizing current pulses (calibration: −30/+15 pA, 5 pA steps, 400 ms; ***B***) used to construct the *I–V* plot shown in ***C*** and to determine the value of R_in_. ***D***, Adapting instantaneous firing, obtained by injecting twice the rheobase current for 1 s. ***E***, Spontaneous firing at resting *V*_m_ (*V*_m_ rest). ***F***, Action potential evoked by a short depolarizing current (3 ms, +800 pA). ***G***, Voltage sag and rebound depolarization generated by hyperpolarizing current injection (500 ms, −150 pA) and blocked by *I*_h_ blocker ZD7288 (30 μm).

We examined the intrinsic membrane properties displayed by pc-nNOS neurons in brain slices from Nos1-Cre^ER^;Ai9 mice (Fig. 2; Table 1; *n* = 10 neurons). These cells were characterized by high R_in_ values (852.8 ± 51.8 MΩ), high membrane time constant (τ, 27.2 ± 2.0 ms), and high excitability (rheobase current, 28.3 ± 4.0 pA), with even small positive current injections leading to sustained firing. Consistent with the general physiology of BLA SOM^+^ neurons (Wolff et al., 2014), pc-nNOS neurons were not fast spiking (instantaneous firing rate, 23.2 ± 1.8 Hz) and showed relatively broad spikes (half-width, 0.75 ± 0.04 ms). Additionally, pc-nNOS cells exhibited very depolarized resting *V*_m_ (−39.7 ± 2.4 mV), often resulting in spontaneous firing (Fig. 2*E*). Finally, when hyperpolarizing currents were injected, pc-nNOS neurons displayed a depolarizing sag and rebound depolarization (Fig. 2). Both responses were mediated by the hyperpolarization-activated cationic current (*I*_h_) because they were abolished by the *I*_h_ blocker ZD7288 (30 μm; sag ratio: control, 0.813 ± 0.031; ZD7288, 1.076 ± 0.081; rebound amplitude: control, 5.9 ± 1 mV; ZD7288, −4.5 ± 3.3 mV; both *p* = 0.048, paired *t* test; *n* = 4; Fig. 2*F*).

**Table 1:** Electrophysiological responses of pc-nNOS neurons

Electrophysiological parameter	Mean ± SEM (*n* = 10)
R_in_ (MΩ)	852.8 ± 51.8
Membrane τ (ms)	27.2 ± 2.0
Membrane capacitance (pF)	32.8 ± 3.1
Rheobase current (pA)	28.3 ± 4.0
Instantaneous firing rate (Hz)	23.2 ± 1.8
Adaptation index	0.62 ± 0.05
CV ISI	0.429 ± 0.071
Rebound depolarization amplitude (mV)	9.6 ± 1.4
Rebound depolarization area (mV × s)	36.5 ± 0.8
Sag ratio	0.915 ± 0.010
Spike half-width (ms)	0.75 ± 0.04
Spike amplitude (mV)	80.6 ± 1.6
fAHP (mV)	16.5 ± 1.2
Threshold potential (mV)	−31.6 ± 0.8
*V*_m_ rest (mV)	−39.7 ± 2.4

Abbreviations: R_in_: input resistance; CV ISI: coefficient of variation of the interspike interval (calculated on the instantaneous firing rate); fAHP: fast after-hyperpolarization; V_m_ rest: resting V_m_.

### Projection pattern and synaptic connectivity of paracapsular type I nNOS^+^ neurons

**Figure 3.**
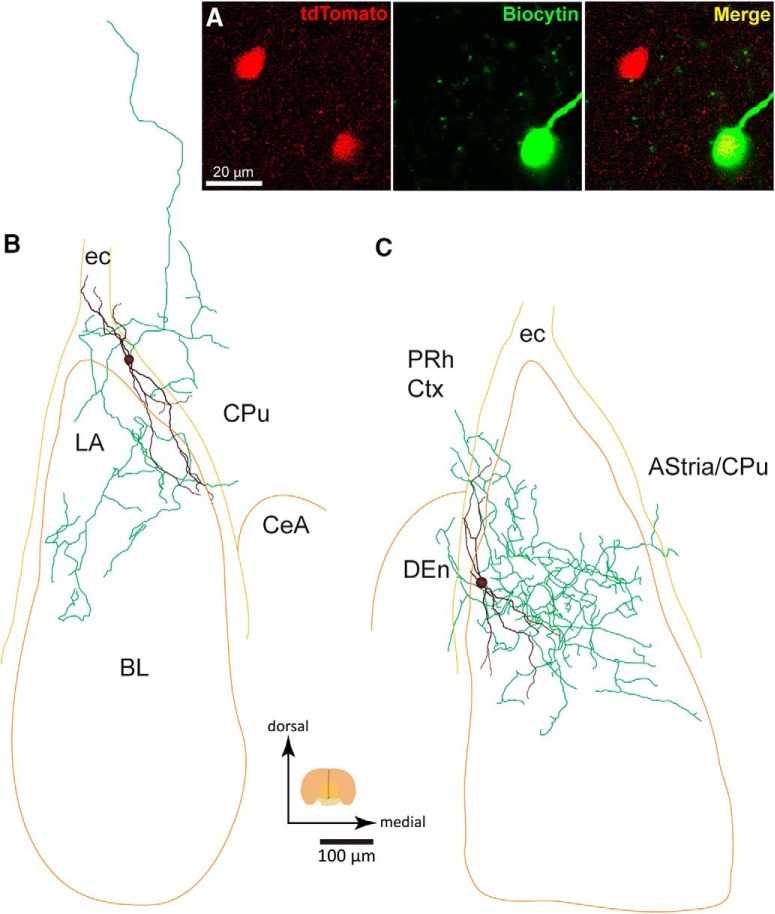
Axonal and dendritic arborization of pc-nNOS neurons. ***A***, Biocytin-labeled pc-nNOS neuron coexpressing tdTomato. ***B***, Neurolucida reconstruction from a pc-nNOS cell (MB131202_1) with dendrites (black) running parallel with the intermediate capsule and axon (green) innervating both the BLA and the caudate–putamen (CPu). ***C***, Neurolucida reconstruction from another pc-nNOS cell (MB151113_2) with dendrites (black) mostly running parallel with the external capsule and axon (green) innervating mostly the BLA, but also the dorsal endopiriform claustrum (DEn), the perirhinal cortex (PRh Ctx), and the amygdalostriatal transition area (AStria)/caudate–putamen (CPu). CeA, Central amygdala; ec, external capsule. Data are presented as the mean ± SEM.

Thus, pc-nNOS neurons are highly excitable because they display high R_in_ values, low rheobase currents, and depolarized *V*_m_ values, suggesting that even small depolarizing synaptic inputs can elicit action potentials in these cells. To clarify the involvement of pc-nNOS cells in the BLA microcircuit, recorded neurons were filled with biocytin to allow *post hoc* anatomical examinations (Fig. 3*A*). Biocytin-filled pc-nNOS cells revealed bitufted dendrites, mainly running parallel to the external capsule. The projection pattern of pc-nNOS neurons was examined with immunofluorescence in 13 cells in which the axon was filled with biocytin. The majority of pc-nNOS cells (10 of 13 cells) innervated the BLA complex. Notably, many pc-nNOS (8 of 13 cells) also sent axonal branches outside the BLA, specifically to the endopiriform claustrum (7 of 13 cells), perirhinal (6 of 13 cells), and piriform (4 of 13 cells) cortices. Additionally, one cell innervated the ectorhinal and temporal association cortices, one cell innervated the amygdalostriatal transition area/caudate–putamen, and one cell innervated the caudate–putamen. This suggests that pc-nNOS cells are not interneurons, because they do not exclusively innervate the BLA, but also innervate extra-amygdaloid regions.

Two pc-nNOS cells were further processed for DAB and reconstructed (Fig. 3*B*,*C*). One of them displayed a dendritic length of 1555.4 μm. Its axon innervated the BLA (2912.14 μm) as well as the caudate–putamen (1954.04 μm; cell MB131202_1; Fig. 3*B*). The other one displayed a dendritic length of 1253.45 μm and innervated the BLA more densely (8077.08 μm). However, shorter axonal branches also targeted the amygdalostriatal transition area/caudate–putamen (185.57 μm), the dorsal endopiriform claustrum (519.97 μm), and the perirhinal cortex (558.8 μm; MB151113_2; Fig. 3*C*). Both cells (together with the other 11 cells observed with fluorescence microscopy) did not show a dense local axonal plexus in the BLA like other NPY^+^ cells (neurogliaform cells; Mańko et al., 2012). Additionally, their axon terminals did not usually form perisomatic basket-like formations (as observed in basket cells; Bienvenu et al., 2012; Vereczki et al., 2016), suggesting that the majority of postsynaptic targets could be dendrites. Thus, pc-nNOS neurons modulate the BLA but also extra-amygdaloid regions.

**Figure 4.**
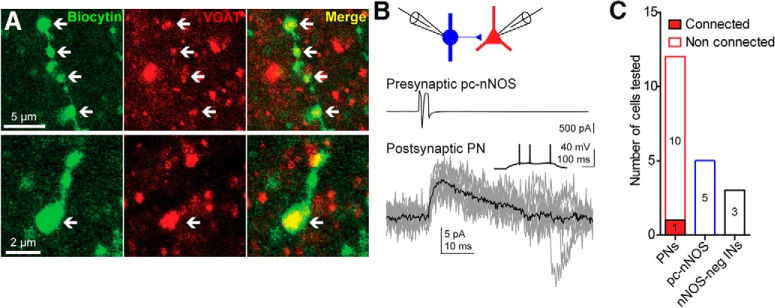
VGAT expression and connectivity of pc-nNOS cells. ***A***, Top, VGAT immunoreactivity of biocytin-filled axonal varicosities of a pc-nNOS neuron (arrows). Bottom, VGAT immunoreactivity in a biocytin-filled bouton from another pc-nNOS cell (arrow). ***B***, Dual whole-cell recording (voltage clamp) showing a presynaptic pc-nNOS neuron functionally connected to a postsynaptic principal neuron (PN). Top, Schematic showing the dual whole-cell recording configuration. Middle, Action current evoked in the presynaptic pc-nNOS. Bottom, uIPSC recorded in the postsynaptic PN (holding potential, −40 mV; gray, overlap of 10 sweeps repeated every 10 s; black, average of the 10 sweeps). Inset, Stereotypical PN firing evoked by 500-ms-long, +100 pA current injection in the postsynaptic cell held at −65 mV. ***C***, Rate of connectivity between a pc-nNOS cell and BLA cells. One of eleven nearby PN cells received a uIPSC, whereas no uIPSC could be recorded in five nearby pc-nNOS cells or three nearby nNOS-negative interneurons.

Immunofluorescence staining for VGAT of sections containing biocytin-filled axons revealed that pc-nNOS boutons are VGAT^+^ (*n* = 2 cells; Fig. 4*A*), confirming that these cells are GABAergic. To study their output synaptic connectivity, we performed paired whole-cell recordings with pc-nNOS as presynaptic neurons and nearby (within ∼100 μm distance) BLA cells as postsynaptic. Firing of a pc-nNOS cell evoked a detectable unitary synaptic current in only 1 of 11 principal neurons (Fig. 4*B*). The unitary synaptic response amplitude was 7.8 pA, its 20–80% rise time was 1.4 ms, and the monoexponential fitted decay time constant was 29.5 ms. The outward current polarity (recorded with normal intracellular with 4 mm Cl^−^) and its kinetic suggest its identity as a GABAergic unitary IPSC (uIPSC). We could not detect any postsynaptic response in five pairs with another pc-nNOS as postsynaptic. Likewise, no postsynaptic response was evoked in three nearby nNOS/tdTomato^−^ interneurons. Thus, in striking contrast to neurogliaform cells, which are also NPY^+^ and display a high connection probability (77%; Mańko et al., 2012), pc-nNOS appear to connect only sparsely with nearby BLA principal cells. Collectively, these data show that pc-nNOS neurons represent a distinctive GABAergic neuron type, because they appear different in terms of electrical, anatomical, and connectivity properties from interneuron types described so far.

### Sleep activates pc-nNOS neurons

Next, we asked in which behaviors and brain states pc-nNOS neurons of the BLA could be activated. In the neocortex, nNOS^+^ neurons have been shown to be inactive after a period of wakefulness, but active after a period of spontaneous sleep or sleep following SD (Gerashchenko et al., 2008; Morairty et al., 2013). Notably, SD prior to sleep appears to be crucial for the activation of nNOS^+^ neurons (Dittrich et al., 2015). Since SD is associated with emotional imbalance (Baglioni et al., 2010) and heightened amygdala responsiveness to salient stimuli (Yoo et al., 2007), a similar pattern of pc-nNOS activation could mean that these neurons track the emotional component of sleep homeostasis.

**Figure 5.**
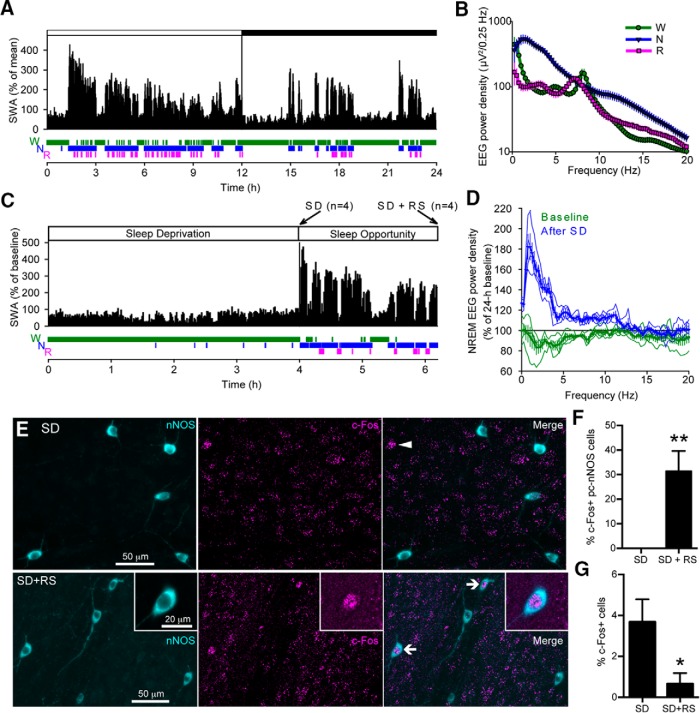
pc-nNOS neurons are activated during sleep. ***A***, The 24 h profile of EEG SWA (EEG power between 0.5 and 4.0 Hz, displayed as the percentage of mean 24 h baseline; white bar, 12 h light period; dark bar, 12 h dark period) recorded in the frontal cortex and below the distribution of sleep–wake stages (W, wakefulness; N, NREM sleep; R, REM sleep) from a representative mouse. Note, as expected, that sleep predominates, and SWA shows a typical decline during the 12 h light period. ***B***, EEG power spectral density during waking, NREM sleep, and REM sleep shown for the frontal EEG (*n* = 7). Note the state-dependent differences in cortical activity. ***C***, Top, Representative profile of SWA during the 4 h SD and subsequent sleep opportunity/RS in one individual mouse. Bottom, The distribution of sleep–wake stages. Mice in the SD group were killed at the end of SD at ZT4 (*n* = 4), while the remaining mice in the SD+RS group (*n* = 4) were killed after the sleep opportunity. ***D***, EEG spectral density in NREM sleep (displayed as a ratio of the mean 24 h baseline) during the sleep opportunity after SD (*n* = 4). Note the typical increase in SWA relative to the corresponding baseline interval after a period of prolonged waking. Thin lines represent the power density from single mice, whereas thick lines represent the mean power density from all four mice. ***E***, Top panels, Confocal stack (*z*-stack, 29 μm) showing lack of c-Fos immunoreactivity in pc-nNOS cells after SD. A median filter was applied (*x*–*y* radius, 5 pixels). Arrowhead, A c-Fos^+^ cell immunonegative for nNOS. Bottom panels, Confocal stack (*z*-stack, 31 μm) showing c-Fos immunoreactivity in two pc-nNOS cells (arrows) following SD+RS. A median filter was applied (*x–y* radius, 5 pixels). Insets, Magnification of one the c-Fos^+^ pc-nNOS cells (*z*-stack, 5 μm; no filtering was applied). ***F***, Quantification of c-Fos expression in pc-nNOS neurons. No pc-nNOS neuron expressed c-Fos following SD, whereas 31.4 ± 16.4% were c-Fos^+^ after subsequent RS (*n* = 4 per condition). ***G***, Quantification of c-Fos expression in paracapsular Nissl-stained cells. Overall, c-Fos^+^ neurons were more abundant following SD (3.7 ± 1.1%) than during the subsequent RS (0.6 ± 0.5%, *n* = 4 per group) **p < 0.01; *p < 0.05. Data are presented as the mean ± SEM.

To investigate whether the activity of pc-nNOS neurons in the amygdala is associated with vigilance state, we performed chronic sleep EEG recordings in eight mice. As expected, during baseline the animals slept predominantly during the light period, and both the typical declining trend of EEG slow-wave activity (SWA) and characteristic vigilance state-dependent differences in EEG spectra were apparent (Fig. 5*A*,*B*; Huber et al., 2000). To determine the activity of pc-nNOS neurons in relation to sleep–wake state mice were subjected to a 4 h SD, beginning at light onset. The SD was successful, with, on average, 97.98 ± 2.51% of time spent awake during the 4 h period. While one group was killed immediately after SD (SD group, *n* = 4 mice), ensuring animals were continuously awake until the moment of perfusion (see Materials and Methods), a second group of mice (SD+RS group, *n* = 4 mice) were allowed a 1.5–2 h sleep opportunity (group average, 1.81 ± 0.15 h) before perfusion. During this interval, the animals were awake for only 5.86 ± 4.13% of the total recording time, while 94.14 ± 4.13% of the interval was spent asleep (89.07 ± 4.78 and 12.48 ± 0.97 min of NREM and REM sleep, respectively). As is typical for early sleep after sleep deprivation, NREM EEG spectral power in slow frequencies during the RS interval were consistently above corresponding baseline values, with the maximal increase observed <4 Hz (Fig. 5*D*). To determine the activation of pc-nNOS neurons, we examined the expression of c-Fos (a marker of neuronal activation; Sheng and Greenberg, 1990) in pc-nNOS^+^ cells in both groups of mice (SD and SD+RS; Fig. 5*E*). Strikingly, we detected no c-Fos^+^ pc-nNOS in mice killed immediately after SD (*n* = 4 brains; 21 ± 3 nNOS neurons counted per brain) In contrast, the percentage of c-Fos^+^ pc-nNOS neurons was significantly higher in SD+RS mice (*p* = 0.0088, unpaired *t* test; *n* = 4 brains; 22 ± 3 nNOS neurons counted per brain; Fig. 5*F*).

Notably, the effect of SD and RS is highly specific for pc-nNOS neurons, because the proportion of c-Fos^+^ neurons (regardless of their neurochemical identity) in the paracapsular region was higher after SD and lower after RS (*p* = 0.0494, unpaired *t* test; *n* = 4 per brains per condition; 223 ± 48 neurons counted per SD brain; 197 ± 22 neurons counted per SD+RS brain; Fig. 5*G*). This finding is in line with previous reports (Cirelli et al., 1995; Semba et al., 2001). These results define a relationship between a specific identified neuron type of the amygdala, namely GABAergic nNOS neurons adjacent to the external capsule, and the vigilance state.

### Dorsal raphe 5-HT neurons innervate pc-nNOS neurons

It has been proposed that the brain state-dependent activity of cortical nNOS^+^ type I cells is powerfully controlled by inhibition exerted by neuromodulators released in arousal states, such as 5-HT, acetylcholine, noradrenaline, and histamine (Kilduff et al., 2011). Conversely, sleep-promoting peptides and hormones have been suggested to promote the recruitment of cortical nNOS^+^ type I cells (Kilduff et al., 2011). Indeed, the release of 5-HT from dorsal raphe neurons is highly dependent on the sleep–wake cycle (Portas et al., 2000). Specifically, the firing of raphe neurons and, as a consequence, extracellular forebrain 5-HT levels are low during sleep and high during spontaneous wakefulness (Portas et al., 1998; Sakai, 2011) or SD (Bjorvatn et al., 2002).

**Figure 6.**
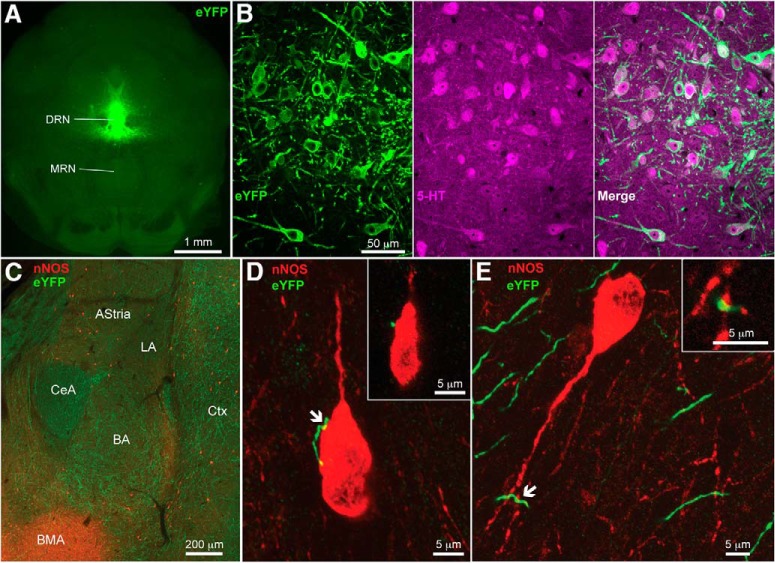
Dorsal raphe 5-HT neurons innervate pc-nNOS neurons. ***A***, Cre-dependent expression of an anterograde tracer in the DRN of SERT-Cre mice ***B***, Confocal stack (*z*-stack, 5.61 μm) showing selective expression of eYFP in 5-HT-immunopositive neurons in the dorsal raphe nuclei. MRN, Median raphe nuclei. ***C***, nNOS immunoreactivity and innervation by dorsal raphe 5-HT neurons in the amygdaloid complex. The external paracapsular region displays prominent innervation. ***D***, Confocal stack (*z*-stack, 11.1 μm) showing axonal varicosities from a dorsal raphe 5-HT neuron juxtaposed to a pc-nNOS neuron soma (arrow). Inset, Magnification of the somatic apposition (single optical section, 0.37 μm thickness). ***E***, Confocal stack (*z*-stack, 5.49 μm) showing an axonal varicosity from a dorsal raphe 5-HT neuron juxtaposed to a pc-nNOS neuron dendrite (arrow). Inset, Magnification of the dendritic apposition (single optical section, 0.37 μm thickness).

Based on this evidence, we hypothesized that dorsal raphe 5-HT neurons target pc-nNOS cells. To this end, we traced the axons of dorsal raphe 5-HT neurons by injecting the viral vector AAV2-EF1a-DIO-EYFP in the dorsal raphe of SERT-Cre mice (Fig. 6*A*). This resulted in selective expression of enhanced yellow fluorescent protein (eYFP) in dorsal raphe 5-HT neurons (with 100% of eYFP^+^ cells also expressing 5-HT, *n* = 3 brains; Fig. 6*B*). eYFP^+^ axons innervated the amygdaloid complex, including the BLA (Fig. 6*C*). Although the LA displayed relatively sparse eYFP^+^ axons, the pericapsular area, where pc-nNOS are located, displayed stronger eYFP^+^ innervation. Examining sections double labeled for eYFP and nNOS from three transfected brains, we consistently found eYFP^+^ axonal varicosities in apposition with pc-nNOS somata (Fig. 6*D*) or dendrites (Fig. 6*E*). These observations suggest that pc-nNOS cells could be modulated by 5-HT released by dorsal raphe neurons.

### 5-HT inhibits pc-nNOS neurons

**Figure 7.**
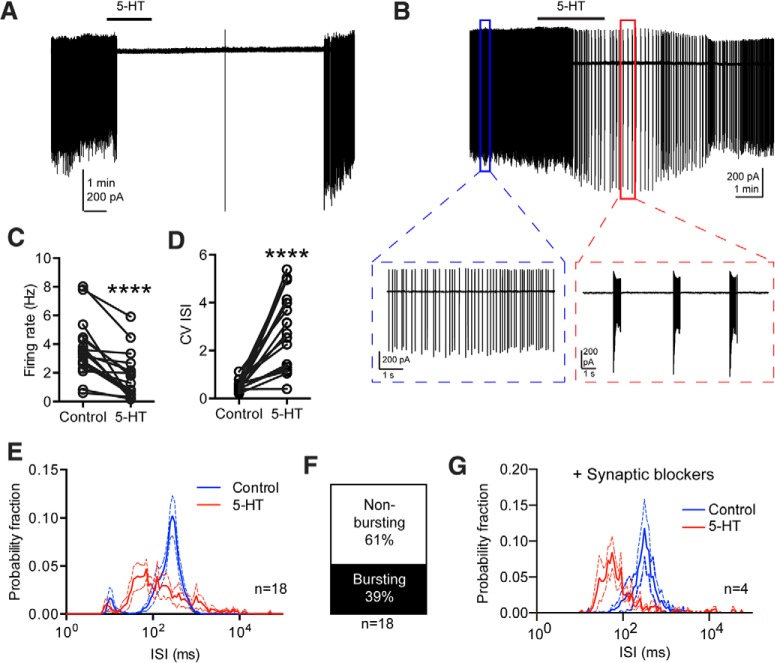
5-HT inhibits pc-nNOS neurons. ***A***, Representative cell-attached recording from a pc-nNOS neuron (voltage-clamp mode) inhibited by bath application of 5-HT (50 μm). In this cell, 5-HT did not trigger burst firing. ***B***, Representative cell-attached recording from a pc-nNOS neuron (voltage-clamp mode) in which bath application of 5-HT elicited a reduction in both firing rate and burst firing. Insets, Magnified examples of tonic firing in control conditions and burst firing upon bath application of 5-HT (50 μm). ***C***, Significant decrease in firing rate promoted by 5-HT (from 3.6 ± 1.6 Hz to 1.6 ± 0.4 Hz; *p* < 0.0001, paired *t* test; *n* = 18). ***D***, Significant increase in firing irregularity (measured by the CV of the ISI: from 0.5 ± 0.06 to 2.9 ± 0.4; *p* < 0.0001, paired *t* test; *n* = 18) caused by 5-HT. ***E***, 5-HT application enhances the burstiness of pc-nNOS neurons: the peak of the ISI histogram (in Log scale) shifts to the left (*n* = 18). ***F***, 5-HT triggered spike bursts in only 7 of 18 pc-nNOS neurons. The remaining neurons displayed a reduction in firing rate only upon 5-HT application. ***G***, In four cells displaying bursts upon 5-HT application, 5-HT was reapplied in the presence of synaptic blockers (10 μm NBQX, 50 μm D-APV, and 10 μm SR95531). In these conditions, 5-HT still triggered bursting (the peak of the Log ISI histogram shifted to the left), suggesting that synaptic inputs are not necessary for bursting activity. ****p < 0.0001. Data are presented as the mean ± SEM.

To test the above-mentioned possibility, electrophysiological and pharmacological experiments were performed. We recorded pc-nNOS neurons in a cell-attached configuration (*n* = 18). As reported above, pc-nNOS neurons fired spontaneously in control conditions. Bath application of 50 μm 5-HT produced a significant reduction in firing rate (from 3.6 ± 1.6 to 1.6 ± 0.4 Hz; *p* < 0.0001, paired *t* test; Fig. 7*A–C*), suggesting an inhibitory effect of 5-HT. Furthermore, 5-HT enhanced pc-nNOS cell firing irregularity [coefficient of variation (CV) of ISI: 0.5 ± 0.06 in controls; 2.9 ± 0.4 in the presence of 5-HT; *p* < 0.0001, paired *t* test; Fig. 7*D*] and, in a minority of cells (*n* = 7), caused a switch from tonic to burst firing (intraburst ISI range, 25–70 ms; Fig. 7*A*,*E*,*F*). Thus, the action of 5-HT on pc-nNOS is inhibitory, and not excitatory, and contrasts with the previously reported depolarizing effects on other BLA GABAergic neuron populations (Rainnie, 1999; Bocchio et al., 2015).

**Figure 8.**
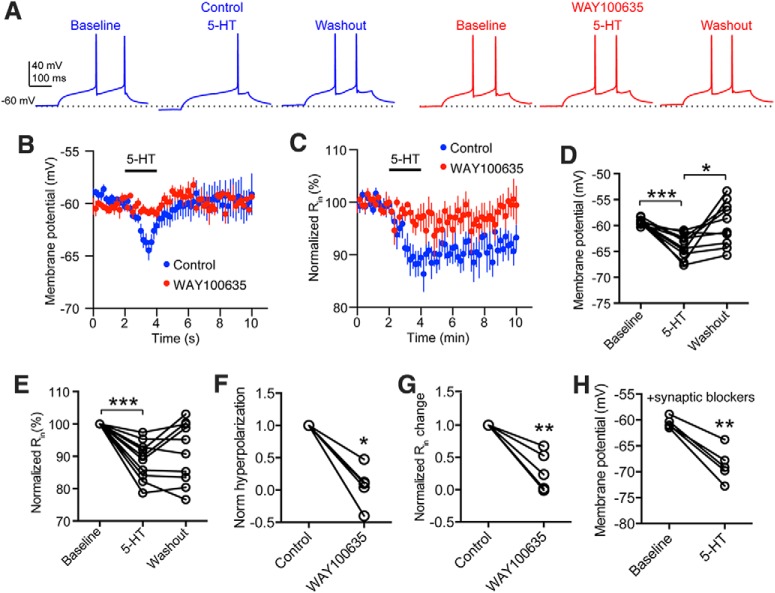
Direct hyperpolarization of pc-nNOS neurons by 5-HT via 5-HT1A receptors. ***A***, Effect of 5-HT on voltage responses to depolarizing current injection of representative pc-nNOS neuron (20 pA, 300 ms) in control conditions (left) and in the presence of 5-HT1A antagonist WAY100635 (10 μm, right). ***B***, Time course of the effect of 5-HT on the *V*_m_ of pc-nNOS neurons (*n* = 10). ***C***, Time course of the effect of 5-HT on the R_in_ of pc-nNOS neurons (*n* = 10). ***D***, 5-HT significantly hyperpolarizes pc-nNOS cells (from −59.3 ± 0.2 to −64 ± 0.7 mV; *p* = 0.001, one-way ANOVA with Bonferroni *post hoc* test; *n* = 10). ***E***, 5-HT significantly reduced the R_in_ of pc-nNOS cells (by 11.3 ± 1.9%; *p* = 0.0006, one-way ANOVA with Bonferroni *post hoc* test; *n* = 10). ***F***, ***G***, 5-HT-evoked hyperpolarization and R_in_ reduction are significantly reduced by 10 μM WAY100635 (*p* = 0.0235 and *p* = 0.0064, respectively, paired *t* tests; *n* = 5). ***H***, 5-HT significantly hyperpolarizes pc-nNOS cells, even in the presence of synaptic blockers (10 μm NBQX, 50 μm D-APV, and 10 μm SR95531; *p* = 0.0017, paired *t* test; *n* = 5), suggesting a direct effect. ***p < 0.001; **p < 0.01; *p < 0.05. Data are presented as the mean ± SEM.

To study the mechanisms through which 5-HT inhibits the firing of pc-nNOS neurons, we performed whole-cell patch-clamp recordings from these neurons in current clamp. Consistent with the inhibition of firing, the application of 50 μm 5-HT elicited membrane hyperpolarization (from −59.3 ± 0.2 to −64 ± 0.7 mV; *p* = 0.001, one-way ANOVA with Bonferroni *post hoc* test; *n* = 10; Fig. 8*A*,*B*,*D*), together with a reduction of the R_in_ (by 11.3 ± 1.9%; *p* = 0.0006, one-way ANOVA with Bonferroni *post hoc* test; *n* = 10; Fig. 8). Both effects were blocked by prior incubation with the 5-HT1A antagonist WAY100635 (10 μm; *p* = 0.0235 and *p* = 0.0064, respectively, paired *t* tests; *n* = 5; Fig. 8). Finally, we confirmed that 5-HT1A-mediated hyperpolarization occurred by a direct effect on pc-nNOS neurons, and was not an indirect network action, because it persisted in the presence of synaptic blockers (10 μm NBQX, 50 μm D-APV, 10 μm SR95531; *p* = 0.0017, paired *t* test; *n* = 5; Fig. 8*H*). Thus, pc-nNOS cells are hyperpolarized by 5-HT via 5-HT1A receptors, in line with the expression of 5-HT1A mRNA in NPY^+^ BLA neurons (Bonn et al., 2013).

Together, these data indicate that pc-nNOS neurons are distinct from other BLA GABAergic cells in that they are hyperpolarized and not depolarized by 5-HT. This hyperpolarization leads to a reduction in firing rate and, in a few cases, to a switch in firing mode. Such 5-HT inhibition could mediate, at least in part, the sleep–wake-dependent modulation of pc-nNOS activity, because extracellular forebrain 5-HT levels are lower during sleep than during wakefulness and SD (Portas et al., 1998; Bjorvatn et al., 2002).

## Discussion

The present study provides novel information on nNOS^+^ type I neurons that surround the BLA. In particular, it describes for the first time the anatomical and physiological properties of these cells, as well as their synaptic connectivity, their activity throughout sleep and wakefulness, and their 5-HT innervation and modulation. We discovered that nNOS^+^ type I neurons are distributed along the boundaries of the BLA, and express SOM, NPY, and NK1. We observed that pc-nNOS neurons are GABAergic, display high intrinsic excitability, relatively broad spikes, voltage sag, and rebound depolarizations, and project both inside and outside the BLA. The activity of pc-nNOS (measured by their c-Fos expression) is low during sleep deprivation and high during subsequent sleep. As a putative cellular mechanism of pc-nNOS cell inhibition during sleep deprivation (and more generally wakefulness), 5-HT, which is known to depolarize GABAergic cells in the BLA, instead hyperpolarizes pc-nNOS cells. Although previous groups reported the presence of putative GABAergic nNOS^+^ neurons of the BLA (McDonald et al., 1993; Usunoff et al., 2006), their physiology and role in behavior remained unexplored.

We discovered that BLA nNOS^+^ neurons can be divided based on the strength of nNOS expression, as previously described for neocortex (Yan et al., 1996). As for neocortex (Magno et al., 2012; Perrenoud et al., 2012), we have termed neurons with strong nNOS expression as type I nNOS^+^ cells and neurons with weak nNOS expression as type II nNOS^+^ cells. We found that nNOS^+^ type I neurons of the BLA coexpress SOM, NPY, and NK1. This combination of neurochemical markers is consistent with the patterns of expression of cortical type I nNOS^+^ cells (Kubota et al., 2011). These data corroborate the notion that the BLA exhibits cortex-like GABAergic neuron diversity patterns (Spampanato et al., 2011; Capogna, 2014). We found that BLA nNOS^+^ type I neurons are preferentially located along the BLA, namely along the external capsule, intermediate capsule, and border between BA and basomedial nucleus. Additionally, examination of biocytin-filled pc-nNOS neurons revealed that their dendrites run in parallel to external or intermediate capsules. The functional reason of this specific localization remains enigmatic. Since external and intermediate capsules are fiber bundles containing axons that originate from several external structures, this distribution might favor their recruitment by glutamatergic axons from distant areas (e.g., sensory thalamus and cortex). Preferential recruitment of these cells could also be facilitated by their high input resistance, low rheobase current, and depolarized resting membrane potential. Overall, these features determine a high intrinsic excitability, with even small excitatory inputs able to elicit action potentials.

Interestingly, SOM^+^ and NPY^+^ neurons located in the external capsule next to the BLA have been shown to be long-range projection neurons, sending their axons to entorhinal cortex (McDonald and Zaric, 2015) and basal forebrain (McDonald et al., 2012). Our study did not demonstrate whether pc-nNOS cells project to these areas or other distant regions, mainly because their axon is likely severed in acute brain slices. However, we found that nNOS type I cells represent virtually all (98.4%) SOM^+^ and NPY^+^ cells of the BLA, suggesting that the SOM^+^ and NPY^+^ pericapsular neurons reported by McDonald et al. (2012) and McDonald and Zaric (2015) could indeed be pc-nNOS cells.

Neurolucida reconstructions of two pc-nNOS cells suggest that these neurons are not pure projection neurons, because they have considerable local projections to the BLA. Nonetheless, for the majority of filled pc-nNOS cells, we detected axonal branches in nearby structures, such as endopiriform claustrum, piriform cortex, and amygdalostriatal transition area/caudate–putamen. In some cases, an axonal branch projected for several hundreds of micrometers into caudate–putamen or cortex. However, we never detected a main, thicker axon typical of other GABAergic long-range projection neurons (e.g., hippocamposeptal and septohippocampal cells; Jinno et al., 2007; Unal et al., 2015b). Importantly, we cannot fully rule out that pc-nNOS cells have a thicker main axon that is myelinated and therefore could not be visualized using fluorescence or light microscopy. Nevertheless, our data keep open the intriguing possibility that pc-nNOS cells coordinate BLA activity with extra-amygdaloid regions.

Using paired whole-cell recordings, we detected a presynaptic pc-nNOS cell functionally connected, likely via a GABAergic connection, to a postsynaptic BLA principal cell. This indicates that BLA principal cells are one of the postsynaptic targets of pc-nNOS neurons. Crucially, pc-nNOS connection probability to principal cells (1 of 11 cellsd) is much lower than the one of other BLA NPY^+^ interneurons (neurogliaform cells; Mańko et al., 2012) or of BLA PV^+^ interneurons (Woodruff and Sah, 2007a). It is not clear whether pc-nNOS cells target BLA GABAergic cells, because we did not detect connections from pc-nNOS cells and nearby pc-nNOS cells (0 of 5 cells) or nearby nNOS-negative interneurons (0 of 3 cells). In addition to clarifying pc-nNOS postsynaptic targets (both in the BLA and in extra-amygdaloid regions), future studies should assess which cellular domains of BLA principal cells are targeted by pc-nNOS neurons. In the BLA, SOM^+^ neurons target distal dendrites of principal neurons, as well as dendrites and cell bodies of interneurons. In line with potential dendritic targeting, our Neurolucida reconstructions revealed that pc-nNOS cells do not innervate BLA or extra-amygdaloid regions with perisomatic basket-like terminals.

pc-nNOS cells are likely to modulate other neurons (i.e., nNOS, NPY, and SOM) not only via GABA release, but also via other neurochemicals. Nitric oxide signaling has been shown to promote long-term potentiation at inhibitory synapses in the LA (Lange et al., 2012), while SOM and NPY appear to hyperpolarize LA principal neurons via G-protein-coupled, inwardly rectifying, potassium channel activation (Meis et al., 2005; Sosulina et al., 2008).

The present study suggests that pc-nNOS cells modulate amygdaloid and extra-amygdaloid neurons in a vigilance state-dependent manner, because our c-Fos data demonstrate that these cells are strongly activated during sleep (at least when sleep follows sleep deprivation). In contrast, pc-nNOS^+^ neurons do not express c-Fos after prolonged wakefulness. To our knowledge, our results represent the first demonstration of a GABAergic neuron type of the amygdala that dichotomously changes its activity as a function of the sleep–wake cycle. Thus, selective sleep activation of nNOS^+^ type I neurons appears to be more widespread and not restricted only to the cortex (Gerashchenko et al., 2008; Morairty et al., 2013; Dittrich et al., 2015).

In agreement with previous findings (Semba et al., 2001), we show that the overall neuronal activation in the paracapsular area is higher after sleep deprivation than after recovery sleep (i.e., a pattern of activation that is the opposite of the one of pc-nNOS neurons). This observation suggests cell type-specific, and not broad, sleep activation in the BLA. Importantly, it is not clear whether all nNOS^+^ type I neurons along or inside BLA boundaries are equally inhibited by 5-HT and are activated by recovery sleep following sleep deprivation. In this study, we limited our quantification to pc-nNOS neurons adjacent to the external capsule to match the location of our patch-clamp recordings.

5-HT has been proposed to be one of the neuromodulators promoting the inhibition of nNOS type I cells in neocortex (Kilduff et al., 2011; Tricoire and Vitalis, 2012). Our study corroborates this hypothesis, because we detected axons from dorsal raphe 5-HT neurons innervating pc-nNOS cells. In addition, electrophysiological experiments demonstrate that 5-HT hyperpolarizes pc-nNOS cells via 5-HT1A receptors. As this hyperpolarization was associated with a decrease in R_in_, it likely arises from the opening of a K^+^ conductance, as described in hippocampal neurons (Andrade and Nicoll, 1987). Interestingly, striatal nNOS interneurons are also inhibited by 5-HT, but this effect is mediated by another class of serotonin receptors (5-HT2C; Cains et al., 2012). Furthermore, in a subset of cells 5-HT also altered the pc-nNOS the firing mode of neurons from tonic to bursting. This bursting physiology in response to membrane hyperpolarization resembles effects previously reported in striatal low-threshold spike interneurons (Dehorter et al., 2009; Beatty et al., 2012), cells that are also SOM^+^, NPY^+^, and nNOS^+^ (Kawaguchi, 1993; Ibáñez-Sandoval et al., 2011). However, this might originate from different mechanisms because pc-nNOS neurons do not display a low-threshold spiking phenotype. The effect exerted by 5-HT provides a putative cellular mechanism that could explain, at least in part, the activity of pc-nNOS neurons across sleep and wakefulness. Their 5-HT-mediated inhibition, an effect previously proposed by Kilduff et al. (2011), could be prominent during sleep deprivation, when 5-HT release from raphe neurons is high, compared with NREM and REM sleep, when 5-HT release is lower (Portas et al., 1998).

It is unlikely that 5-HT is the only neurotransmitter released during wakefulness and arousal that suppresses pc-nNOS neuron activity. For example, paracapsular SOM^+^ and NPY^+^ neurons have been shown to coexpress the muscarinic type 2 acetylcholine receptor (McDonald and Mascagni, 2011), implying an inhibitory action of acetylcholine. Since pc-nNOS neurons express NK1 receptors, a putative source of neuromodulatory excitatory drive on these cells is the NK1 agonist substance P. Importantly, NK1^+^ neurons have been shown to regulate anxiety and reward processing (Gadd et al., 2003; Truitt et al., 2009). Future studies should establish whether pc-nNOS neuron activity is high during sleep due to intrinsic membrane properties or also because of stronger excitatory inputs. These inputs could include peptides and hormones released during sleep, such as adenosine (as proposed by Kilduff et al., 2011), or glutamatergic axons contained in external/intermediate capsules.

In summary, our work turns the spotlight on a novel BLA GABAergic cell type that is activated by sleep and inhibited by wakefulness and 5-HT, and establishes a link between BLA circuits and sleep–wake history. Given the crucial involvement of the BLA in conditioned fear and anxiety (Tovote et al., 2015), this discovery is particularly compelling because sleep deprivation has been shown to impact both emotional phenomena (Graves et al., 2003; Silva et al., 2004). In the future, intersectional genetic approaches will allow selective tagging of nNOS^+^ type I neurons (He et al., 2016), for instance by taking advantage of their SOM, NPY, or NK1 expression. In addition to facilitating their targeting for electrophysiological recordings, these strategies could also permit specific manipulation of BLA nNOS^+^ type I cells during behavior, which could probe their precise role in sleep and emotion regulation.

## Synthesis

Reviewing Editor: Zoltan Nusser, Institute of Experimental Medicine<br />Decisions are customarily a result of the Reviewing Editor and the peer reviewers coming together and discussing their recommendations until a consensus is reached. When revisions are invited, a fact-based synthesis statement explaining their decision and outlining what is needed to prepare a revision will be listed below. For some manuscripts transferred from JNeurosci, a decision is made to accept the manuscript without peer review and a brief statement to this effect will follow.

The authors have performed a number of experiments during their revision and addressed most concerns of the reviewers. Both reviewers felt that the link between the in vitro and in vivo part is rather weak. I agree with this concern, but both the in vitro and vivo experiments seem to be performed thoroughly and do not seem to have major shortcomings. Although it is widely used, the c-Foc expression analysis still holds uncertainties, which is also true in this study.
